# Mistakes and Pitfalls Associated with Two-Point Compression Ultrasound for Deep Vein Thrombosis

**DOI:** 10.5811/westjem.2016.1.29335

**Published:** 2016-03-02

**Authors:** Tony Zitek, Jamie Baydoun, Salvador Yepez, Wesley Forred, David E. Slattery

**Affiliations:** * University of Nevada School of Medicine, Department of Emergency Medicine, Reno, Nevada; † University Medical Center of Southern Nevada, Department of Emergency Medicine, Las Vegas, Nevada

## Abstract

**Introduction:**

Two-point compression ultrasound is purportedly a simple and accurate means to diagnose proximal lower extremity deep vein thrombosis (DVT), but the pitfalls of this technique have not been fully elucidated. The objective of this study is to determine the accuracy of emergency medicine resident-performed two-point compression ultrasound, and to determine what technical errors are commonly made by novice ultrasonographers using this technique.

**Methods:**

This was a prospective diagnostic test assessment of a convenience sample of adult emergency department (ED) patients suspected of having a lower extremity DVT. After brief training on the technique, residents performed two-point compression ultrasounds on enrolled patients. Subsequently a radiology department ultrasound was performed and used as the gold standard. Residents were instructed to save videos of their ultrasounds for technical analysis.

**Results:**

Overall, 288 two-point compression ultrasound studies were performed. There were 28 cases that were deemed to be positive for DVT by radiology ultrasound. Among these 28, 16 were identified by the residents with two-point compression. Among the 260 cases deemed to be negative for DVT by radiology ultrasound, 10 were thought to be positive by the residents using two-point compression. This led to a sensitivity of 57.1% (95% CI [38.8–75.5]) and a specificity of 96.1% (95% CI [93.8–98.5]) for resident-performed two-point compression ultrasound. This corresponds to a positive predictive value of 61.5% (95% CI [42.8–80.2]) and a negative predictive value of 95.4% (95% CI [92.9–98.0]). The positive likelihood ratio is 14.9 (95% CI [7.5–29.5]) and the negative likelihood ratio is 0.45 (95% CI [0.29–0.68]). Video analysis revealed that in four cases the resident did not identify a DVT because the thrombus was isolated to the superior femoral vein (SFV), which is not evaluated by two-point compression. Moreover, the video analysis revealed that the most common mistake made by the residents was inadequate visualization of the popliteal vein.

**Conclusion:**

Two-point compression ultrasound does not identify isolated SFV thrombi, which reduces its sensitivity. Moreover, this technique may be more difficult than previously reported, in part because novice ultrasonographers have difficulty properly assessing the popliteal vein.

## INTRODUCTION

Deep vein thrombosis (DVT) is difficult to diagnose clinically,^1^ and thus requires imaging for diagnosis. Although the ultimate gold standard for diagnosis is contrast venography,^2^ ultrasound performed by a technologist and interpreted by a radiologist is the current test of choice for diagnosis of DVT in the emergency department (ED).^3^ With the potential to save both time^4^ and money, emergency physician (EP)-performed DVT ultrasound offers an attractive alternative to reliance on radiology department-performed ultrasound imaging. Indeed, the American College of Emergency Physicians (ACEP) supports EP-performed DVT ultrasound training, as it now considers DVT ultrasound one of the core emergency ultrasound applications.^5^

At least 12 studies have evaluated EP-performed ultrasound studies to assess for DVT.^4^,^6^–^16^ While some of these studies have found both the sensitivity and specificity for EP-performed DVT ultrasounds to be greater than 90%,^7^,^11^,^12^,^16^ estimates of the sensitivity and specificity across these studies are inconsistent. Two systematic reviews^17^,^18^ and meta-analysis^19^ have evaluated EP-performed DVT ultrasonography, and have found the sensitivity and specificity to be in the mid to high 90s, but they lament the heterogeneity of the studies used in their analyses, and in some cases, the relatively few number of operators with a likely high degree of expertise.

Among the studies cited above that have evaluated EP-performed DVT ultrasounds, there is a great degree of variability in the technique used. Some studies have used a two-point compression technique,^4^,^6^,^8^,^9^,^11^ in which only two locations are tested for compressibility – one in the groin to assess the common femoral vein and one in the popliteal fossa to test the popliteal vein. Other studies have used a three-point compression technique, which in addition to the common femoral and popliteal veins, assesses the superior femoral vein (SFV) at a single location for compressibility.^10^,^13^,^15^ (Remember that despite its name, the SFV is a deep vein.) At least one study has evaluated EPs performing duplex ultrasounds of the entire leg,^16^ and at least one study evaluated EPs performing compression ultrasound of the entire proximal leg,^7^ excluding calf veins.

There are limited data comparing these various techniques, but one recent study found excellent results with the simplest of these techniques, the two-point compression technique. This study used 47 physicians, including emergency medicine (EM) attendings, EM residents, and residents rotating in the ED from other services. After only a 10-minute training session, they were able to use two-point compression ultrasound to achieve a sensitivity of 100% and a specificity of 99%.^11^ Given the apparent ease by which this technique could be learned and the purported accuracy of this technique, if these results could be replicated, the two-point compression technique would clearly be the preferred technique in the ED. However, although data from radiology departments supports the use of a two-point compression technique,^20^–^22^ to our knowledge no study using EPs as the operators has been able to replicate these results.

Our study was designed to attempt to replicate the results of the above-named study. We aimed to test the accuracy of the two-point compression technique for diagnosis of lower extremity proximal DVT as performed by EM residents with no prior formal training in this technique compared to the gold standard performed by ultrasound technologists and interpreted by radiologists. We also performed an analysis of our EM residents’ ultrasound videos to determine if the ultrasound images were adequate and to assess for common errors that might be made when performing two-point compression ultrasound. Finally, we sought to characterize the potential impact on ED length of stay (LOS) if EPs were to make disposition decisions based on their bedside DVT ultrasound.

## METHODS

### Study Design

This was a prospective diagnostic test assessment of a convenience sample of ED patients suspected of having a lower extremity DVT. This study was approved by our hospital’s institutional review board.

### Study Setting and Population

This study was performed in the adult ED (annual census of approximately 83,300) of an academic, county, tertiary-care referral facility with a three-year EM residency.

### Study Protocol

EM residents in our facility had no previous formal training in the two-point compression technique for diagnosing proximal lower extremity DVTs. All EM residents received approximately a two-hour training session that included a lecture on how to perform two-point compression ultrasound, practice on a human model, and a competency test with videos asking the residents to identify whether or not a DVT was present. As this study took place during two separate academic years, two separate training sessions were performed. The first trained and tested all EM residents in the program during the spring of 2013. The second session trained and tested the incoming intern class in July 2013. The format of the training sessions was identical.

Two-point compression ultrasound requires that the ultrasonographer identify the common femoral vein near the inguinal crease and the popliteal vein in the popliteal fossa. The a priori criteria for considering an ED ultrasound study positive were if either the vein would not compress completely from wall to wall or if an echogenic focus (representing a thrombus) was identified. EM residents were instructed to strictly follow the two-point compression method, and not to compress in other areas or use any supplemental methods for identifying DVT such as Doppler color flow or “augmentation.” Augmentation is accomplished by squeezing the leg just distal to the site being examined with Doppler to help analyze flow through the vein. All ED scans were done with a standard 7.5 MHz linear probe on the Mindray M7.

Patients were eligible for inclusion in the study if they were at least 18 years old and suspected of having DVT with a radiology department-performed DVT ultrasound ordered. We excluded patients if they had any of the following: a known DVT, a DVT within the previous six months, a DVT ultrasound within the last month, or a radiology department DVT ultrasound performed immediately prior to enrollment. We also excluded patients who were pregnant or in law enforcement custody.

Eligible patients were approached by research assistants or EM residents and shown a brief video explaining DVT and the purpose and process of the study. Written, informed consent was obtained, and then the ordering physician recorded basic patient demographics.

The subject underwent a two-point compression ultrasound by one of the EM residents; the resident determined whether or not the patient had a DVT and if so, which vein was involved. EM residents were instructed to save the videos of their scans, and to record their start and finish times on the data collection form. In some cases the ordering physician was also the resident who performed the two-point compression ultrasound. For the purposes of this research, orders for bilateral ultrasound constituted two separate studies. Subsequently, each subject underwent ultrasonography performed by a technologist from the radiology department, and interpretation was done by an attending radiologist. The radiology ultrasound technician and radiologist were blinded to the ED ultrasound results. Residents were prohibited from telling the patient whether the test was positive or negative, and no change in clinical care was permitted based on the resident’s ultrasound study. The gold standard test was performed on all patients regardless of the EM resident ultrasound result.

The technique used by our ultrasound technologists involves a minimum of six points of compression: at the common femoral vein, just distal to the inguinal ligament, at three points along the SFV (proximal, middle, and distal), and at the popliteal vein and the posterior tibial vein. Any area that is noncompressible is considered positive for venous thrombosis. Any echogenic focus within the vein lumen is also considered a positive study. We grouped acute and chronic thrombi together in this study. Also, this study was concerned with identification of proximal lower extremity DVTs, so thrombi identified in the calf veins by the radiologists were considered negative studies. The technologists in our institution also perform Doppler examination at all six sites to look for flow, and they perform augmentation.

The study was conducted from May 10, 2013, to July 5, 2014. There are no EM residents in our department from 6 pm on Tuesdays until 2 pm on Wednesdays, so the study was temporarily suspended during those hours.

### Outcome Measures

The primary outcome measure was the test performance characteristics for identification of a proximal lower extremity DVT by the two-point compression technique performed by EM residents as compared with a gold standard of radiology department ultrasounds. We also performed an analysis of the EM residents’ ultrasound videos to assess them for adequacy, and to determine what errors the residents made when performing the compression ultrasounds. Finally, we sought to determine the EM resident-performed ultrasound’s potential impact on ED LOS by comparing the time from ultrasound order placement to completion of the resident’s ultrasound, compared to time of order placement to the official reading by the radiologist.

### Data Analysis

We stored and analyzed data collected for this study in a Microsoft Excel (Version 14, Microsoft, Redmond, WA) spreadsheet. Based on the size of previous EP-performed DVT ultrasound studies,^4^,^6^–^16^ all but one^16^ of which have had fewer than 200 patients, we aimed to enroll 400 patients. This would make our study the largest study of EP-performed two-point compression ultrasound.

## RESULTS

From May 10, 2013, through July 5, 2014, 32 EM residents performed compression ultrasound on 234 patients with a total of 288 ultrasounds performed. (Patient characteristics are shown in Table 1). In all cases, the gold standard test was performed. Flow of patients is shown in Figure.

### Accuracy

The results of the ultrasounds performed for this study are shown in Table 2. Of the 288 ultrasounds performed, 28 cases were deemed to be positive for DVT by the radiology ultrasound. Sixteen of the 28 were correctly identified by the residents with two-point compression as true positive DVTs. Among the 260 cases deemed to be negative for DVT by radiology ultrasound, 10 were falsely thought to be positive by the residents using two-point compression. Overall, the EM residents had a sensitivity of 57.1% (95% [CI 38.8–75.5]) and a specificity of 96.1% (95% CI [93.8–98.5]) for identification of proximal lower extremity DVT. This led to a test accuracy of 92.4% with a positive predictive value of 61.5% (95% CI [42.8–80.2]) and a negative predictive value of 95.4% (95% CI [92.9–98.0]). The positive likelihood ratio is 14.9 (95% CI [7.5–29.5]) and the negative likelihood ratio was 0.45 (95% CI [0.29–0.68]).

Thirty-two unique ultrasound operators contributed to this study. There was a large range in the number of studies performed by each resident. Three residents performed only one ultrasound, and one resident performed 51 ultrasounds. Eleven residents performed at least 10 ultrasounds. The results of the ultrasounds for each operator can be seen in the appendix.

### Analysis of Resident Videos

The videos of the EM residents’ ultrasounds in which the radiologist’s interpretation differed from the resident’s were reviewed. The total number of legs in which this was the case was 22 (10 false negatives and 12 false positives). Four of these videos could not be reviewed because they were not properly recorded and/or saved by the resident.

In two of the 22 discrepancies, the resident did not achieve adequate images of the common femoral vein. In one of these two, this appeared to be because the resident confused a lymph node with the common femoral vein.

In eight of the 22 cases, the resident’s incorrect interpretation could be attributed to inadequate visualization of the popliteal vein. In the majority of those videos, the popliteal vein was not visible and it is not clear what structure the resident thought was the popliteal vein. However, in some of those cases, it appeared that a superficial vein was likely being confused with the popliteal vein. In one of those videos, the resident thought what was likely the tibial nerve was actually the popliteal vein with a hyperechoic thrombus.

In three of the 22 analyzed videos, the residents made other types of mistakes. In two, the residents obtained adequate visualization of the common femoral vein, but did not press hard enough to appropriately test for compressibility. In the third, the resident obtained adequate visualization of the common femoral vein, and upon review of the video, it appeared that part of the vein was not compressible. The resident, however, incorrectly interpreted the images and determined that there was no thrombus.

Finally, five of the 22 videos appeared to have adequate images of both the common femoral and popliteal veins despite being interpreted as having a DVT by the radiologist. In four of those cases, there was an isolated thrombus in the SFV, so there was no mistake on the resident ultrasonographer’s part since evaluation of the SFV is not part of the two-point compression technique. In the other case, there appeared to be adequate compression of both the common femoral and popliteal veins upon our review of the videos, but the radiologist’s report read “suspicious for small partial thrombus,” as he or she felt there was a very small part of the vessel that was not collapsible.

### Speed

The median (and interquartile range [IQR]) time for EM residents to complete a two-point compression ultrasound was four minutes (IQR two to eight minutes, minimum less than one minute, maximum 24 minutes). The ED ultrasounds were completed in a median of 84 minutes (IQR 62 to 119 minutes, minimum 15 minutes, maximum 756 minutes) before the radiology ultrasound report was made available to the EPs.

## DISCUSSION

The sensitivity for two-point compression ultrasound in this study was significantly lower than in some of the previous studies of EP-performed DVT ultrasound,^7^,^11^,^12^,^16^ including the study upon which we modeled this study.^11^ There are several possible reasons for this.

First, the operators in our study may have been less experienced or less skilled at ultrasound. Several of the previous studies used operators who were highly trained attendings,^6^ had “extensive experience,”^8^ or who had a 30-hour training course.^16^ However, the study by Crisp, et al.^11^ had 47 operators with varying experience, many of whom had no prior experience, similar to the operators in our study. Thus, our results contradict those of the Crisp study in that it does not appear that one can become competent at two-point compression ultrasound after a brief training session. Indeed, our ultrasound video analysis demonstrated that at least 13 of the 22 discrepancies between the radiologist’s and EM residents’ ultrasounds could be attributed to an EM ultrasonographer error that might have been avoided with more intensive training, expanded training on how to avoid the pitfalls found in this study, or more experience. If these 13 cases had been evaluated by a more experienced ultrasonographer we would have achieved sensitivity and specificity that would more closely resemble those of some previous studies of EP-performed DVT ultrasound.

Another reason that the sensitivity in our study may have been lower than in previous EP-performed DVT ultrasound studies is that the two-point compression technique may be inferior to other DVT ultrasound techniques. The previous studies showing the highest sensitivities and specificities for EP-performed DVT ultrasounds used more thorough techniques,^7^,^12^,^16^ such as complete proximal leg ultrasound^7^ or whole leg ultrasound (including calf veins).^16^ The exception to this was the study by Crisp, et al.,^11^ which used a two-point compression technique and found a sensitivity of 100% and a specificity of 99%. At this point, that study appears to be an outlier. Indeed, our results were similar to those from a recent study of two-point compression performed by inexperienced operators on intensive care unit (ICU) patients (sensitivity 63%, specificity 97%).^23^

Logically, it would make sense that an ultrasound that does not specifically evaluate the SFV would have low sensitivity for identifying DVTs in it, but several studies from the 1990s suggest that a two-point compression technique is adequate,^21^,^22^,^24^–^26^ and the study by Crisp, et al.^11^ seems to provide EM-specific support for this. The idea behind this technique is that isolated SFV thrombi are rare.

However, more recent data suggest isolated SFV thromboses occur with some regularity, which would make the two-point compression technique undesirable. Indeed, a recent study found that 5.5% of proximal lower extremity DVTs were isolated to the SFV,^27^ suggesting that a two-point compression technique should theoretically not achieve a sensitivity greater than the mid-90s, even with perfect ultrasound technique. Additionally, a study of two-point compression on ICU and intermediate care patients found six isolated SFV thromboses out of 12 patients who had DVTs.^23^

ACEP’s Emergency Ultrasound Imaging Criteria Compendium supports two-point compression ultrasound, stating that the evaluation of the SFV “is not a primary focus of the standard lower extremity EUS [emergency ultrasound studies] evaluation.”^28^ Based on the results of our study and other recent data discussed above, we do not support this statement. We suggest using a protocol that routinely evaluates the SFV.

We admit that the sensitivity and specificity reported in this paper do not represent the greatest sensitivity and specificity that could be achieved using the two-point compression technique with more experienced operators. This begs the question: how many ultrasounds does one have to perform to achieve proficiency in compression ultrasound?

In one prior study of EP-performed compression ultrasounds, sensitivity was initially mediocre but became 100% after having performed ultrasounds on three patients.^10^ We, however, doubt that proficiency can be attained so easily. In our study, reanalyzing the data only for residents who had already performed three compression ultrasounds for our study did not produce a statistically significant improvement in sensitivity or specificity. The recalculated sensitivity was 66.7% (95% CI [47.8–85.5]) and the specificity was 95.5% (95% CI [92.4–98.5]). Thus, while there is likely a learning curve for performing compression ultrasound, our study did not amass enough data to determine when an operator becomes proficient at performing this test; performing three ultrasounds does not appear to be sufficient.

Compared to some previous studies, one aspect that made our study unique was that we had the EM residents record videos of their ultrasounds, so that we could assess the images on which they based their interpretations. We are unaware of any previous study that has specifically analyzed EP-performed DVT ultrasounds for the purpose of finding common mistakes that might be made by novice ultrasonographers. This analysis produced several interesting findings.

First, the most common error made by EM resident ultrasonographers was inadequate visualization of the popliteal vein. Given the high frequency at which errors appear to occur at the popliteal vein, ultrasound educators should take heed of this to specifically target avoiding this error. ACEP’s Emergency Ultrasound Criteria Compendium does not list this as a potential pitfall of DVT ultrasound^28^, when in fact our data suggest it is the most common error.

Second, our analysis of the EM residents’ ultrasounds suggests that DVT ultrasound training should emphasize how to distinguish lymph nodes, nerves, and DVTs. Training should also emphasize how to determine how much pressure to apply when compressing the veins. Although the training session we provided did include some information about these topics, it may have been insufficient.

Finally, the four cases in which the EM residents missed an isolated SFV thrombus bring us back to the point that two-point compression ultrasound may not be sufficient for diagnosis of lower extremity DVT.

Lastly, this study confirmed the findings of other studies^4^,^15^ that have found that EP-performed compression ultrasound is rapid (generally less than five minutes based upon our results) and has the potential to reduce the patient’s time to disposition and time to treatment significantly. This makes further research in this field imperative to determine an ultrasound protocol to evaluate for DVT that has better test characteristics and is still rapid.

## LIMITATIONS

This study had several limitations that are important to consider when interpreting the results. First, we used a convenience sampling of patients and that may have resulted in some patients who would have been more difficult to ultrasound (for example, because of obesity) not getting enrolled.

This was a single-center study, which limits the generalizability, but we had 32 operators, which is more than all but two of the previous EP-performed DVT ultrasound studies.^10^,^11^ The fact that the ultrasonographers were all novices also limits the generalizability of the results in some ways, but the study was designed in this fashion for two main reasons. First, the study by Crisp, et al.^11^ found excellent results with a heterogeneous group of ultrasonographers, including many who had only received a10-minute training session. While our training was more extensive than in that study, we otherwise copied the design of that study. Second, the use of novice ultrasonographers allowed us to perform an analysis of common mistakes made by the resident ultrasongraphers for educational purposes. The majority of EPs currently practicing did not learn DVT ultrasound during their residencies; therefore, the majority of EPs are novices at DVT ultrasound, and in this sense, it makes our study more generalizable than some of the previous studies.

In our study, one resident performed 51 of the 288 DVT ultrasounds (about 18% of all the ultrasounds). This resident was also an investigator on the study, and so was particularly motivated. Allowing one resident to perform this many ultrasounds could have resulted in inflated sensitivity, and capping the number of ultrasounds allowed by a given resident may have been preferable.

Another limitation to consider was that our study was undersized because of under-enrollment. A large contributing factor in this regard was that our ED implemented a new electronic medical record (EMR) system approximately six months into the data collection period. We relied heavily on research assistants to identify patients for possible enrollment, but the new EMR restricted the access of the research assistants and made it more difficult for them to identify patients for enrollment. The study was ended prior to reaching our enrollment goal because the rate of enrollment dropped dramatically after the implementation of the new EMR system. Nonetheless, 288 ultrasounds were included, which makes this, to the best of our knowledge, the largest study of EP-performed two-point compression DVT ultrasound to date. The additional 112 ultrasounds would have been primarily helpful to assess for the number of ultrasounds it takes for one to become competent at two-point compression ultrasound.

There were some instances in which the residents did not completely follow study protocol. This resulted in some ultrasound videos not being available for our review. Moreover, some residents only recorded their overall start and finish times instead of the time it took to complete each individual leg. In these cases, the time for each leg was recorded as the total time for both legs, when the time actually would be about half that length. This means the overall time to complete a two-point compression ultrasound is likely shorter than seven minutes.

Finally, we recognize that radiology department ultrasounds represent a false gold standard, since as mentioned above, contrast venography actually represents the gold standard test for DVTs. However, given the rarity by which contrast venography is ordered, radiology ultrasound is functionally the gold standard in the ED, and this study would not have been feasible if we chose contrast venography as our gold standard.

## CONCLUSION

Although, compression ultrasound shows promise as a means for EPs to rapidly diagnose proximal lower extremity DVT, the two-point compression method does not identify thrombi isolated to the SFV, which detracts from the method’s sensitivity. Although more experienced operators may be able to achieve higher sensitivities and specificities with two-point compression ultrasound than what we achieved, after video analysis of the ultrasounds performed in this study, it is clear that isolated SFV thrombi may occur and be missed even by a perfectly performed two-point compression technique. Future DVT ultrasound studies should focus on techniques that include an evaluation of the SFV.

Our findings suggest that it is more difficult to become competent at compression ultrasound than previously thought. Emergency ultrasound educators should be aware of this and of the difficulties that novice ultrasonographers have in properly assessing the popliteal vein for DVT.

## Figures and Tables

**Figure:**
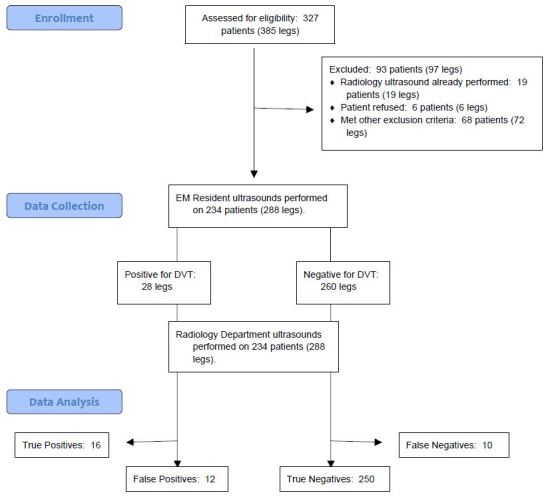
STARD flow diagram demostrating the flow of patients. *EM,* emergency medicine; *DVT,* deep vein thrombosis

**Table 1:** Baseline characteristics of patients.

Category	Value
Age	
Range 18–85 (median 48)	--
Gender	
Male	50.7%
Female	49.3%
Race	
African American	41.0%
Caucasian	34.7%
Hispanic	15.3%
Asian/Pacific Islander	4.2%
Other/Not documented	4.9%
Smoker?	
No	64.6%
Yes	30.6%
Not documented	4.9%

**Table 2:** Results of EM resident-performed 2-point compression ultrasounds and calculated test characteristics.

	Positive radiology ultrasound	Negative radiology ultrasound
Positive ED ultrasound	16	10
Negative ED ultrasound	12	250
Sensitivity, %	57.1 (95% CI [38.8–75.5])	--
Specificity, %	--	96.1 (95% CI [93.8–98.5])
Positive predictive value	61.5% (95% CI [42.8–80.2])	--
Negative predictive value	--	95.4% (95% CI [92.9–98.0])
Positive likelihood ratio	14.9 (95% CI [7.5–29.5])	--
Negative likelihood ratio	--	0.45 (95% CI [0.29–0.68])

*ED*, emergency department
